# Imaging Active Infection *in vivo* Using D-Amino Acid Derived PET Radiotracers

**DOI:** 10.1038/s41598-017-08415-x

**Published:** 2017-08-11

**Authors:** Kiel D. Neumann, Javier E. Villanueva-Meyer, Christopher A. Mutch, Robert R. Flavell, Joseph E. Blecha, Tiffany Kwak, Renuka Sriram, Henry F. VanBrocklin, Oren S. Rosenberg, Michael A. Ohliger, David M. Wilson

**Affiliations:** 1 Department of Radiology and Biomedical Imaging, University of California, San Francisco, CA 94158 USA; 2 Department of Medicine, University of California, San Francisco, CA 94158 USA; 3 Department of Radiology, Zuckerberg San Francisco General Hospital, San Francisco, CA 94110 USA

## Abstract

Occult bacterial infections represent a worldwide health problem. Differentiating active bacterial infection from sterile inflammation can be difficult using current imaging tools. Present clinically viable methodologies either detect morphologic changes (CT/ MR), recruitment of immune cells (^111^In-WBC SPECT), or enhanced glycolytic flux seen in inflammatory cells (^18^F-FDG PET). However, these strategies are often inadequate to detect bacterial infection and are not specific for living bacteria. Recent approaches have taken advantage of key metabolic differences between prokaryotic and eukaryotic organisms, allowing easier distinction between bacteria and their host. In this report, we exploited one key difference, bacterial cell wall biosynthesis, to detect living bacteria using a positron-labeled D-amino acid. After screening several ^14^C D-amino acids for their incorporation into *E. coli* in culture, we identified D-methionine as a probe with outstanding radiopharmaceutical potential. Based on an analogous procedure to that used for L-[methyl-^11^C]methionine ([^11^C] L-Met), we developed an enhanced asymmetric synthesis of D-[methyl-^11^C]methionine ([^11^C] D-Met), and showed that it can rapidly and selectively differentiate both *E. coli* and *S. aureus* infections from sterile inflammation *in vivo*. We believe that the ease of [^11^C] D-Met radiosynthesis, coupled with its rapid and specific *in vivo* bacterial accumulation, make it an attractive radiotracer for infection imaging in clinical practice.

## Introduction

A common clinical problem for practicing infectious disease specialists and radiologists is differentiating active infection from other causes of inflammation. This problem is of critical importance because the treatments for infection and sterile inflammation are diametrically opposed, with antibiotics being indicated in one case and immunosuppression in the other. Definitive diagnosis requires tissue sampling, but the diagnostic accuracy of this procedure can be low, and the associated morbidity may be high, for example in biopsies of the central nervous system (CNS). Noninvasive anatomic imaging techniques, especially computed tomography (CT) and magnetic resonance imaging (MRI) are also frequently used in the workup of acute infection. Although many infections have a characteristic imaging appearance on CT and MRI, it is often impossible to distinguish bacterial infection from other disease entities. For example, active bone infection may look similar on MRI to radiation-induced necrosis, rheumatologic disease or age-related degeneration^1, 2^. Several molecular imaging methods have been applied to this problem, in particular ^111^In or ^99m^Tc white blood cell (WBC) scanning, in which the patient’s own immune cells are radiolabeled and planar gamma camara imaging or single-photon emission computed tomography (SPECT) is performed^3^. Recently, the workup of fever of unknown origin (FUO) has included full-body positron emission tomography (PET) using 2-[^18^F]-fluorodeoxyglucose (FDG), the most common radiotracer applied to oncologic imaging^4^. Like cancer cells, activated immune cells are highly glycolytic, and thus may be detected using FDG-PET. However, lack of specificity is the major problem for both SPECT and PET approaches. Furthermore, these imaging techniques are dependent on host inflammatory responses to infection, which may be reduced or absent in immunosuppressed patients (e.g. chemotherapy, HIV/AIDS, organ transplant) who are most at risk for infection.

In order to overcome these barriers, there has been a long and sustained interest in developing specific probes that can be used to label bacteria *in vivo* during active infection^5^. Many innovative strategies have been reported including radiolabeled antibodies^6^, antimicrobial peptides^7^, antibiotics^8^, specific enzyme ligands^9^, nucleic acids^10^, metabolized compounds^11^ and even bacteriophages^12^. To date, no agent has been accepted into clinical practice for routine differentiation of infection from sterile inflammation.

**Figure 1:**
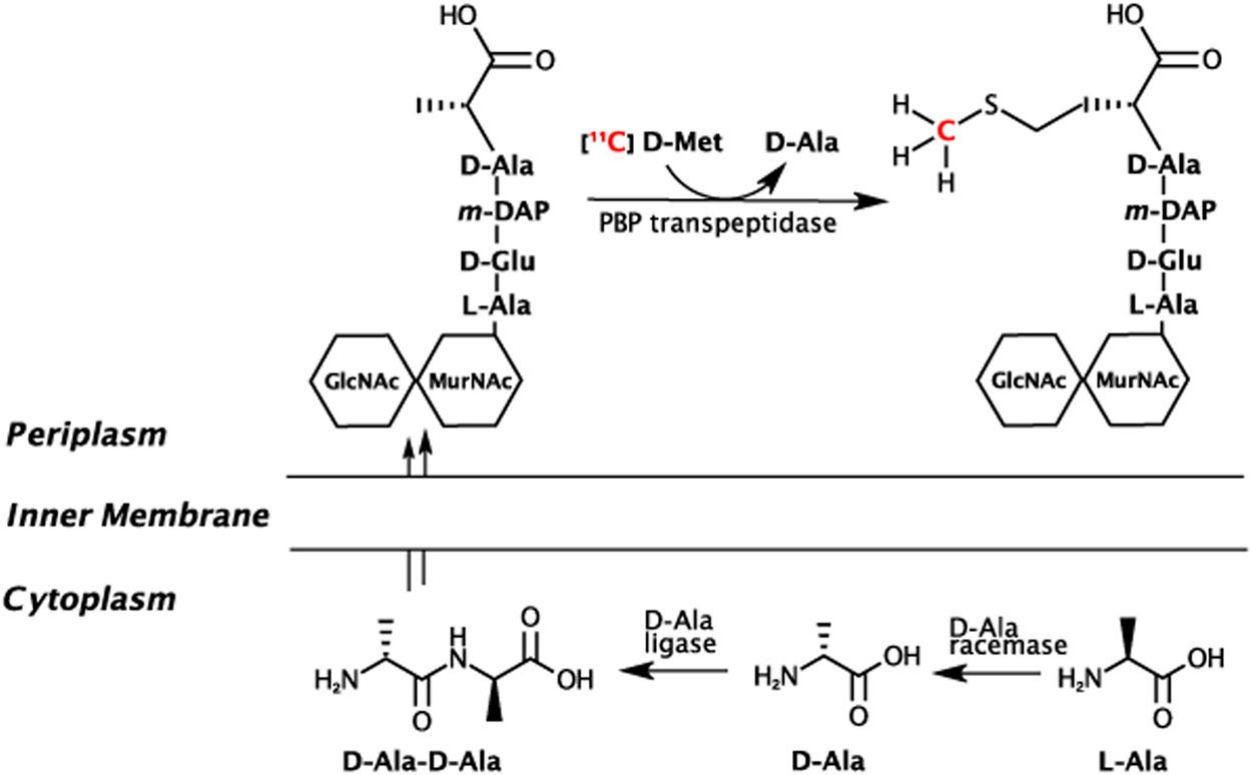
Incorporation of D-amino acids into peptidoglycan via racemase-dependent and racemase-independent pathways. The intracellular processing of D-alanine is contrasted with the perpiplasmic addition of D-methionine, which is “swapped” at the C-terminus of peptidoglycan, mediated by the transpeptidase domains of penicillin binding proteins (PBPs). D-Met may be incorporated into peptidoglycan muropeptides by exogenous administration or by physiologic production, with the latter associated with transition into the stationary phase and downregulation of peptidoglycan synthesis. The putative pathway of ^11^C retention following [^11^C] D-Met administration to infected animals is highlighted in red. GlcNAc = N-acetylglucosamine, MurNAc = N-acetyl- muramic acid, m-DAP = meso-diaminopimelic acid.

Most bacteria produce and incorporate significant amounts of D-amino acids (DAAs), in particular D-Ala and D-Glu, which are used in peptidoglycan synthesis. Peptidoglycan is a strong and elastic polymer of the bacterial wall that maintains cell shape and anchors components of the cell envelope^13^. Interestingly, DAA within peptidoglycan appear to protect the bacterial cell against peptidase and protease attacks and also serve important and specific roles in cell-cell signaling^14^. Many antimicrobial agents (both synthetic and natural products) work by antagonizing the DAA synthesis, dimerization, and incorporation pathway, most notably beta-lactam antibiotics and other cell wall active agents like vancomycin and cycloserine^15^. Remarkably, other DAA are incorporated into peptidoglycan via a mechanism that is independent of the normal biosynthetic pathway, inducing structural alterations^16^. These DAAs include D-Met and D-Phe, which are readily incorporated into *Escherechia coli* muropeptides and likely serve important roles in cell signaling in addition to structural function^14^. In particular D-Met has been shown to be released by a diverse array of stationary phase bacteria, which use it as a specific signal to alter the growth of neighboring cells. Importantly, D-Met released into the media is avidly taken up by bacteria and incorporated into peptidoglycan^14^. D-amino acids have been shown to be powerful agents for *in vitro* labeling of peptidoglycan^17^ and their incorporation is very rapid (~30 sec in *E. coli*) and highly specific^18^ prompting us to consider the possibility of using these compounds *in vivo* for diagnostic imaging (Fig. 1). Adapting methods previously described^19^, we synthesized [^11^C] D-Met and applied it to the detection of both *E. coli* and *S. aureus* bacteria *in vivo*.

## Results

### [^14^C] D-Met is readily accumulated in both *E. coli* and *S. aureus*

**Figure 2:**
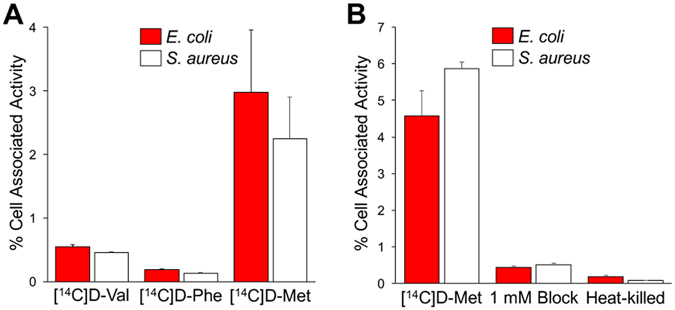
*In vitro* accumulation of ^14^C D-amino acids in *E. coli* and *S. aureus*. (**A**) Panel of ^14^C amino acids studied for relative uptake. (**B**) Dedicated ^14^C D-Met study in *E. coli* and *S. aureus* including incubation with heat-killed organisms and in the presence of 1 mM unlabeled D-methionine.

An *in vitro* screen of [^14^C] D-amino acids was performed in small-volume *E. coli* and *S. aureus* cultures, to identify promising structures for ^11^C or ^18^F incorporation (Fig. 2A). *E. coli* and *S. aureus* were chosen as representative gram-negative and gram-positive bacteria; the pathogenic strains of these organisms are frequent culprits in human infection^20, 21^. Based on a prior report documenting rapid incorporation of DAAs into *E. coli* muropeptides *via* a racemase-independent pathway, [^14^C] D-Val, [^14^C] D-Phe and [^14^C] D-Met were studied^16^. Incorporation of ^14^C was quantified by liquid scintillation counting. This study showed the highest percent cell associated activity for [^14^C] D-Met, which was identified as the best candidate for PET tracer development. Importantly, this screen also showed D-Met was readily incorporated by both *E. coli* and *S. aureus*. Of note, D-Ala and D-Glu were considered as potential cell-wall labeling agents, since they are both components of native peptidoglycan. However, pursuing PET versions of these molecules had significant drawbacks: significant *in vivo* defluorination for ^18^F versions of alanine^22, 23^, modest enantiomeric excess (48% ee; 74%-L, 26%-D) for reported [^11^C] L-Ala asymmetric synthesis^24^, and lack of homology between reported ^18^F versions of glutamate and the native amino acid^25, 26^. The development of stable, high enantiomeric excess PET versions of these metabolites is an important goal for future studies.

The behavior of [^14^C] D-Met was further studied in competition (blocking) experiments, evaluated in heat-killed bacteria, and compared to that of [^14^C] L-Met. Heat-killed bacteria did not accumulate [^14^C] D-Met and the uptake of the radiotracer was effectively blocked by co-incubation with 1 mM concentrations of unlabeled D-Met in normal bacteria (Fig. 2B), confirming specificity to metabolically active organisms (p < 0.05). Comparing bacterial uptake of [^14^C] D-Met and [^14^C] L-Met yielded discrepant results, depending on the media used. In F12 media (low amino-acid concentration), there was no significant difference between [^14^C] D-Met and [^14^C] L-Met accumulation (p > 0.05) (Supp. Fig. 1A **)**. In contrast, marked selectivity for [^14^C] D-Met over [^14^C] L-Met was observed in lysogeny broth (LB) (p < 0.05) (Supp. Fig. 1B). The reason for this difference is unknown and may be related to the lower amino acid content in F12 media. However, the D/L selectivity in LB observed *in vitro* was recapitulated by *in vivo* studies as discussed subsequently.

### [^11^C] D-Met can be produced rapidly and efficiently via an automated radiosynthesis

**Figure 3:**
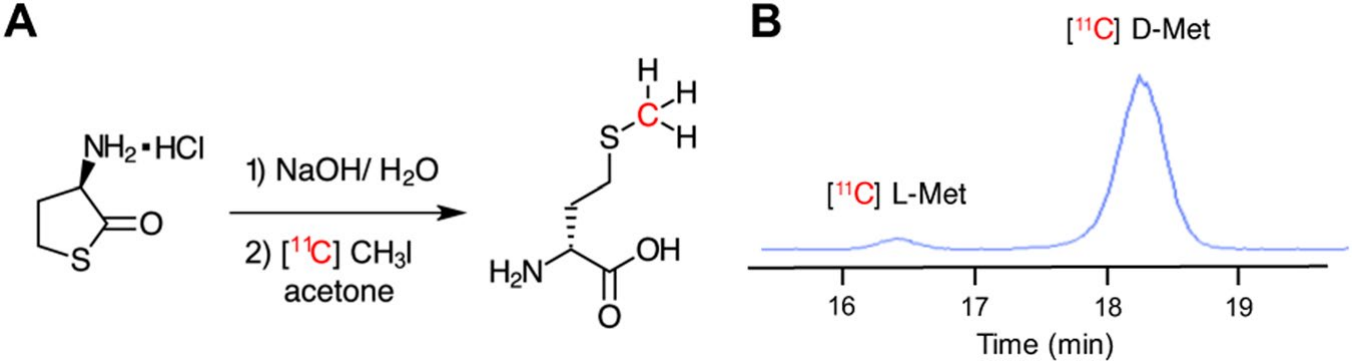
Radiosynthesis of [^11^C] D-Met from D-homocystinethiolactone precursor. (**A**) Synthetic scheme. (**B**) Chiral stationary-phase HPLC showing enhanced enantiomeric excess synthesis of [^11^C] D-Met.

Several radiosyntheses of [^11^C] L-Met have been described, first by Langstrom *et al*. in 1997 using a Na/NH_3_ reduction of an S-benzyl protected homocysteine precursor^27^. For our study, we chose the method most frequently applied to automated radiopharmaceutical preparation of [^11^C] L-Met for patient studies, namely [^11^C] CH_3_I methylation of an L-homocysteinethiolactone precursor^19^. After synthesis of the corresponding D-homocysteinethiolactone, we first optimized this radiosynthesis for enhanced enantiomeric excess of [^11^C] D-Met via gas-phase produced [^11^C] CH_3_I on a GE FX/C Pro™. Base-catalyzed hydrolysis of D-homocysteinethiolactone is prone to racemization, as has been previously reported^28^. As the central hypothesis of this study was *in vivo* differential identification of bacteria using the D enantiomer, we explored different hydrolysis conditions to optimize enantiomeric excess (%ee) of the final product (Supp. Fig. 2 and Supp. Table 1). We found that a final concentration of 3.3 mM NaOH gave us the highest enantiomeric excess of [^11^C] D-Met. For our optimized protocol, gas-phase produced [^11^C] CH_3_I was reacted with the D-thiolactone precursor and NaOH in an acetone/H_2_O mixed solvent for 2 minutes at 100 °C, followed by quenching with AcOH. For subsequent studies using these conditions (n = 4), we isolated [^11^C] D-Met in 21 min (EOB) in a 20.3 ± 1.2% non-decay corrected (41.6% corrected) radiochemical yield with a specific activity of 4.5 ± 3.2 Ci/μmol and 92.6% ± 2.4% D-enantiomer (85% enantiomeric excess). For all [^11^C] D-Met *in vivo* studies reported chiral HPLC confirmed greater than 90% D-enantiomer (Fig. 3).

### [^11^C] D-Met PET can differentiate active bacterial infection from sterile inflammation *in vivo*

**Figure 4:**
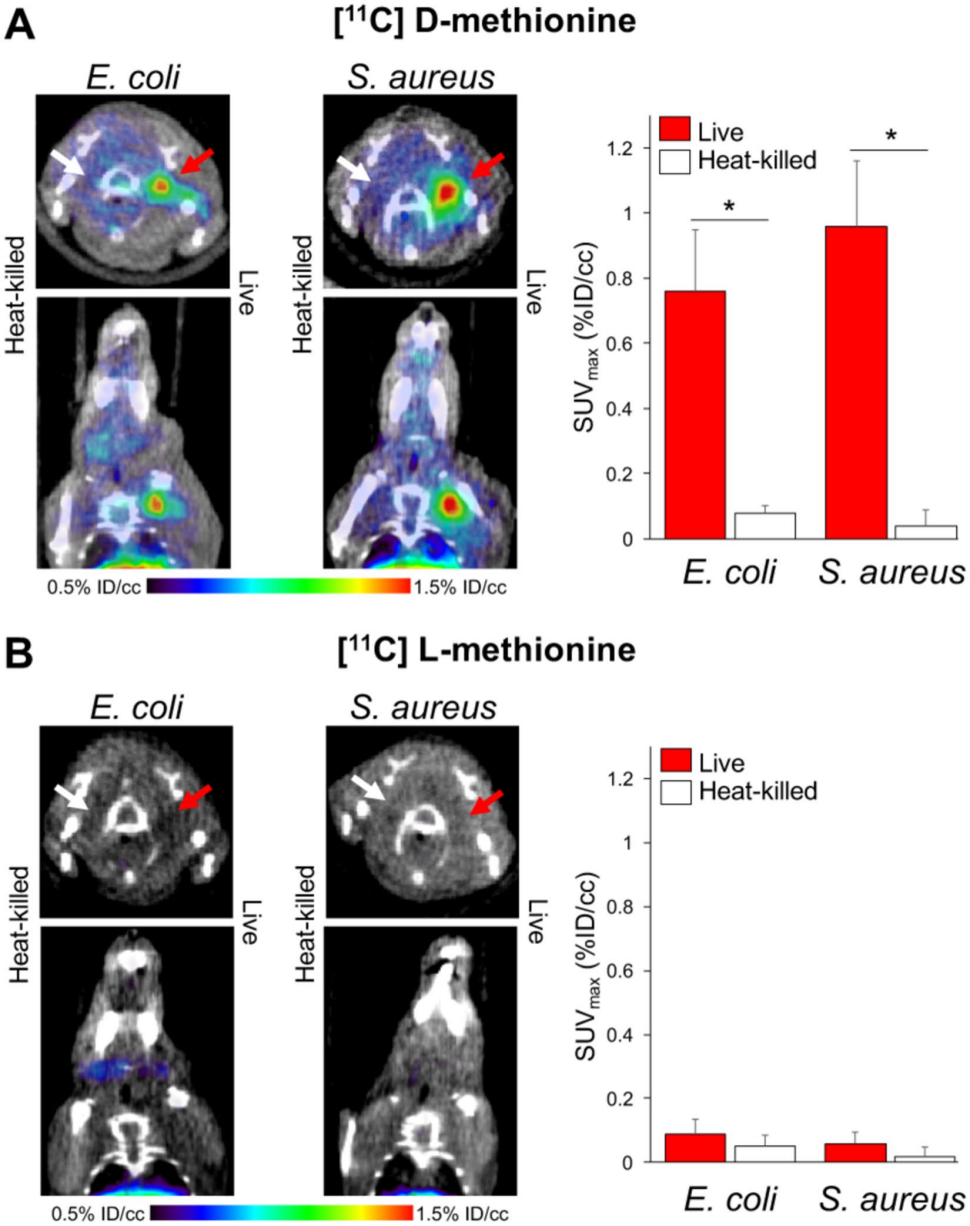
*In vivo* studies using [^11^C] D-Met and [^11^C] L-Met a murine myositis model (n = 4 for each condition studied). In all cases the site of live bacterial inoculation is denoted by a red arrow, while the site of 10X heat-killed bacterial inoculation is denoted by a white arrow. (**A**) [^11^C] D-Met studies in *E. coli* and *S. aureus*-infected animals. Representative images show marked uptake in areas corresponding to live bacterial injection (left deltoid), in contrast to sterile inflammation (right deltoid) and normal muscle. ROI analysis of *E. Coli* and *S. aureus* [^11^C] D-Met cohorts, corrected using normal muscle uptake. (**B**) [^11^C] L-Met studies in *E. coli* and *S. aureus*. There is no observable difference in signals from the left (infected) and right (sterile inflammation) deltoid muscles. ROI analysis of *E. Coli* and *S. aureus* [^11^C] L-Met cohorts, showing no significant difference in accumulation between infection and sterile inflammation.

**Figure 5:**
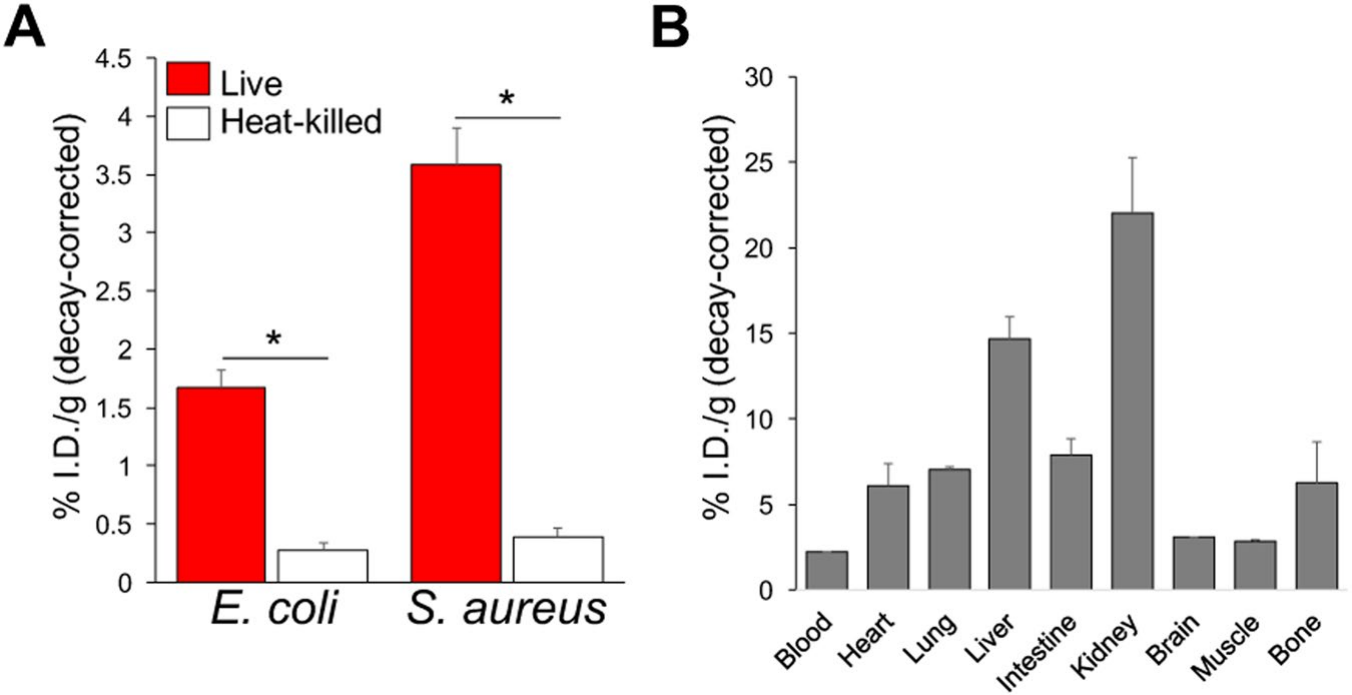
*Ex vivo* analysis of both infected and normal mice, obtained via tissue harvesting and gamma-counting. In all cases mice injected intravenously with [^11^C] D-Met were sacrificed at 70 minutes. (**A**) Analysis of harvested deltoid muscles, performed immediately following the imaging studies described in this manuscript. The deltoid muscles corresponding to inoculation with live bacteria were compared with the contralateral side (heat-killed). (**B**) The biodistribution of [^11^C] D-Met was also studied in a separate cohort of normal CBA/J female mice. The highest accumulation was observed in the kidneys and liver, similar to previously reported findings for [^11^C] L-Met^34^.

Based on the promising results from our *in vitro* studies, we next investigated whether [^11^C] D-Met was a suitable imaging agent for differentiating bacterial infection from sterile inflammation *in vivo*, using a murine myositis model similar to that previously reported^11^. Confirmations of the validity of this model included both repeating *in vivo* results using 2-[^18^F]-fluorodeoxysorbitol (FDS), and postmortem tissue homogenization and plating to confirm the presence of living bacteria. Mice were inoculated with live *E. coli* or *S. aureus* in the left deltoid muscle and a 10-fold higher burden of heat-killed bacteria in the contralateral deltoid (sterile inflammation). After the infection was allowed to progress for 12 hours, the mice were imaged by μPET-CT using a single time-point study similar to clinical protocols. [^11^C] D-Met rapidly and specifically accumulated in the infected region-of-interest (ROI), while no detectable accumulation above background was observed in the sterile, inflamed right deltoid (Fig. 4). Spherical ROIs were drawn to quantify the accumulation of D-Met in the infected deltoid, the contralateral sterile inflammation site, and normal muscle. When normal muscle ROI’s were used to normalize the data, dramatic differences between infection and sterile inflammation were seen (Fig. 4B). Uncorrected data showed [^11^C] D-Met produced >2-fold higher counts in the infected ROI as compared to the sterile inflammation site (Supp. Fig. 3), with representative maximum intensity projection (MIP) images shown in Supp. Fig. 4. In contrast, no significant difference was seen between infected versus sterile sites when using [^11^C] L-Met (Fig. 4C,D) (p > 0.05). This ROI analysis was corroborated by biodistribution studies on animals following euthanasia (Fig. 5A).

**Figure 6:**
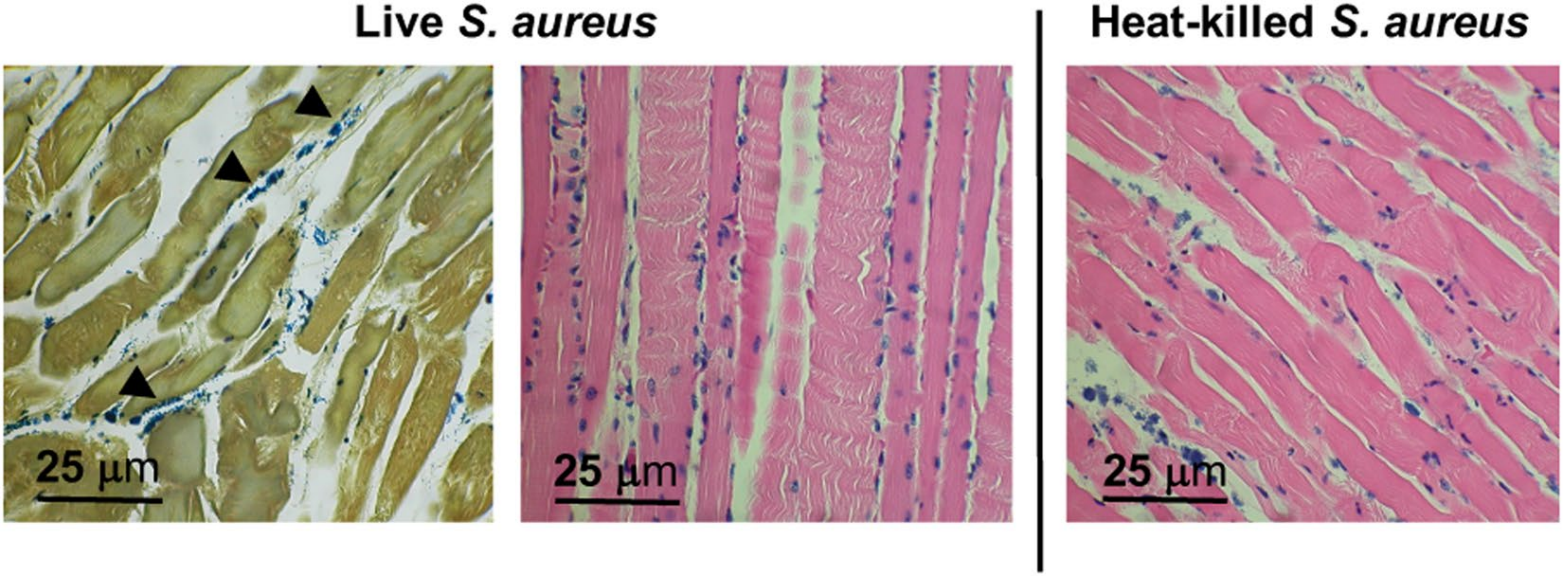
Representative histology for an *S. aureus* infected mouse sampled from deltoid muscle, using Gram-staining (left image) and hematoxylin & eosin (H&E, center and right image). In live inoculations, scattered inflammatory cells and intact bacteria are seen, best identified by Gram-staining and denoted by arrowheads. In heat-killed inoculations (right), several inflammatory cells are present on H&E staining without discernible bacteria. Images are representative of four animals.

Postmortem tissue histology (hematoxylin and eosin, Gram-stain) for both strains of bacteria in mice thighs verified the presence of inflammatory cells and bacteria in the infected thigh, and inflammatory cells without bacteria in the contralateral uninfected thigh. Representative data for *E. Coli* and *S. aureus* inoculations (n = 4 animals studied per organism) are shown in Supp. Fig. 5 and Fig. 6 respectively. We further demonstrated the presence of sterile inflammation in this model using FDG. Since Weinstein *et al*. previously applied FDG to murine *E. coli* infection^11^, we studied a cohort of *S. aureus* infected mice (n = 4, Supp. Fig. 6). We demonstrated concordant results, namely that similar FDG uptake was seen in muscle inoculated with live and 10X heat-killed bacteria (p > 0.05). Both showed increased tracer accumulation versus normal muscle by *ex vivo* analysis (dissection and gamma counting), p < 0.05).

## Discussion

The imaging of infection using bacteria-specific metabolic pathways has tremendous clinical potential. A PET probe for bacterial infection would be most useful in the clinic if it could distinguish bacterial infection (all organisms) from common mimics. Thus, we sought to develop a PET probe that is accumulated by representative gram-negative and gram-positive pathogenic bacteria, to distinguish infection from other CT and MR mimics. Encouraging data are emerging from other groups investigating potentially broad-spectrum maltose and maltodextrin-derived tracers^29–31^, but to date no lead tracer has emerged with wide microorganism sensitivity, facile radiosynthesis, and good *in vivo* stability. Therefore, in this study we developed a D-amino acid derived PET probe that is accumulated by both *E. coli* and *S. aureus* infections, as a starting point for more detailed study of (1) microorganism sensitivity and (2) other D-amino acid derived scaffolds. This strategy has not been previously applied to *in vivo* imaging, despite the numerous antibiotics (for example vancomycin) targeting bacterial cell wall synthesis.

The findings of this study set the stage for additional D-amino acid derived PET tracers. Several studies have indicated that many unnatural D-amino acids are incorporated into bacterial peptidoglycan at a high rate, including work performed in the Bertozzi laboratory, showing the incorporation of R-propargylglycine into *E. coli* peptidoglycan^17^. Recently, Pidgeon *et al*. demonstrated that transpeptidases catalyze the metabolic incorporation of exogenous D-amino acids onto bacterial cell surfaces with vast promiscuity for C-terminus variations^32^. This tolerance for structural differences will facilitate incorporation of ^11^C, ^18^F, and other nuclei into related radiotracers. Eventually, the incorporation of a set of unique tracers could be used to differentiate bacteria and guide treatment decisions in settings where bacterial culture and sensitivity is not immediately available.

A limitation of infection imaging strategies is that human microbiomes contain huge numbers of non-pathogenic bacteria that colonize the internal and external surfaces of human bodies^33^. The presence of these bacteria in our respiratory and gastrointestinal tracts may limit detection of pathogenic bacteria in these locations. Furthermore, some tracers including [^11^C] D-Met have significant background uptake in normal organs, most notably the kidney and liver (Fig. 5B). Fortunately, several of the most challenging infections from a diagnostic standpoint occur in normally sterile environments, for example within the nervous, biliary and musculoskeletal systems. The workup of these conditions might benefit greatly from [^11^C] D-Met, particularly in conjunction with multi-modality imaging (PET-CT and PET-MR).

## Methods

### [^14^C] D-amino acid uptake studies in *E. coli* and *S. aureus*

*E. coli* strain ATCC 25922 and *S. aureus* strain ATCC 12600 were used for all *in vitro* and *in vivo* studies. The *in vitro* studies evaluating both *E. coli* and *S. aureu*s for relative [^14^C] substrate accumulation were prepared identically. Each condition reported was a measure of four replicates containing 20 million bacteria. Bacteria were inoculated in LB broth and grown overnight to OD_600_ = 1.0. The bacteria were then pelleted by centrifugation at 13.2 rpm for 60 seconds. The supernatant was removed, and the pellet was resuspended in 1 mL of F12 media or LB broth. For blocking experiments, four replicates were prepared in the same manner with 20 million bacteria, 100uL of 10 mM D-Met, and 1 mL of F12 or LB broth. The vials were incubated with 0.1 µCi of [^14^C] D-amino acid (Moravek Inc.) at 37 °C for 2 hours. The bacterial suspensions were removed from the incubator and centrifuged at 13.2 rpm for 60 seconds. The supernatant was removed, and 1 mL of ice-cold phosphate-buffered saline (PBS) was added to the samples and vortexed gently for 3 seconds. The suspension was centrifuged at 13.2 rpm for 60 seconds, the supernatant was removed, and the wash cycle was repeated one additional time. Upon removal of the supernatant, 500 μL 1 M NaOH was added to the bacterial pellets. Each pellet was vortexed for 3 seconds and incubated at 37 °C for 5 minutes. A 400 μL aliquot was transferred to a scintillation vial and diluted with 3 mL of MP Biomedicals Ecolyte liquid scintillation cocktail. Samples were counted on a Beckman LS 6500 scintillation counter.

### Radiochemical synthesis of [^11^C] D-Met

[^11^C] CO_2_ was produced in target by the ^14^N(p, α)^11^C nuclear reaction of 17 MeV protons on N_2_ in the UCSF radiopharmaceutical facility. [^11^C] CO_2_ was converted to [^11^C] CH_3_I using the gas-phase method on a GE FX/C Pro automated synthesis module. [^11^C] CH_3_I (g) was transferred from a Porapak N into a glass reactor vial, previously charged with 500 µL of acetone, while maintaining the reactor vial temperature at 0 °C during the transfer. After 3 minutes, 0.2 mg of D-homocysteine thiolactone hydrochloride, dissolved in 100 µL of 20 mM NaOH (aq), were transferred to the reactor vial. The reactor vial was sealed and heated at 100 °C for 2 min. The reactor vial was cooled to 40 °C and acetone is evaporated over the course of 3 minutes. The aqueous reactor vial solution was neutralized with 320 µL of 10 mM acetic acid (previously loaded in V2) and the contents of the reactor vial are transferred through a C18 Sep-Pak® Plus and collected in a vial for use. Enantiomeric excess of the final product was analyzed using a Phenomenex Chirex-33126 D-penicillamine HPLC column (4.6 × 250 mm) with a mobile phase of 30:70 methanol:1 mM copper sulfate at a flow rate of 1 mL/min. Retention times of [^11^C] L-Met and [^11^C] D-Met were 16.4 and 18.2 minutes, respectively.

### Murine Myositis Model

All animal procedures were approved by the UCSF Institutional Animal Care and Use Committee. Veterinary services for the study were provided by the UCSF Laboratory Animal Resource Center (LARC) and all studies were performed in accordance with UCSF guidelines regarding animal housing, pain management, and euthanasia. All mice used were CBA/J females (Jackson Laboratory) aged between 8–10 weeks. Single colonies of *E. coli* or *S. aureus* were placed in LB broth shaking cultures at 37 °C overnight prior to inoculations. Cultures were incubated until they reached OD_600_ of 1.0. 1 mL portions of the culture were heat killed by incubating at 90 °C for 30 minutes. The heat-killed bacteria were then spun down and resuspended in 100 μL of LB broth. Mice were placed under isoflurane anesthesia on a warming pad. Approximately 8 hours before imaging, 100 μL of live bacterial culture and 100 μl of 10X concentrated heat-killed bacteria were injected with tuberculin syringes into the right and left shoulder musculature, respectively. Mice were then removed from anesthesia and allowed to recover and intermittently monitored prior to imaging.

### PET imaging

Under isoflurane anesthesia, a tail vein catheter was placed. For methionine studies, approximately 1 mCi of [^11^C] D-Met or [^11^C] L-Met were injected via the tail vein catheter. The animals were placed on a heating pad to minimize shivering. Mice were allowed to recover, micturate, and at 45 minutes post-injection, placed back under isoflurance anesthesia. At 1 hour post-injection, the animals were transferred to a Siemens Inveon micro PET-CT system (Siemens, Erlangen, Germany), and imaged using a single static 10 min acquisition (60–70 minutes post-injection), followed by micro-CT scan for attenuation correction and anatomical co-registration. Studies using FDG were performed using a similar protocol; 200 μCi of FDG were injected via tail vain catheter and the animals imaged using a static 15 minute acquisition (45–60 minutes post-injection). No adverse events were observed during or after injection of any compound. Anesthesia was maintained during imaging using isofluorane. Upon completion of imaging, mice were sacrificed and biodistribution analysis performed using either harvested deltoid muscle (infected mice) or normal organs (wild-type mice). Gamma counting of harvested tissues (n = 4 per organ or tissue) was performed using a Hidex Automatic Gamma Counter (Turku, Finland).

### Data analysis and statistical considerations

All PET data were viewed using open source Amide software (amide.sourceforge.net). Quantification of uptake was performed by drawing regions of interest over indicated organs on the CT portion of the exam, and expressed as percent injected dose per gram. All statistical analysis was performed using Microsoft Excel. Four data sets were acquired for all *in vitro* and *in vivo* studies (n = 4 in all cases). Data were analyzed using an unpaired two-tailed Student’s t-test. All graphs are depicted with error bars corresponding to the standard error of the mean. Other data including specific activity, radiochemical yield, and % D-enantiomer are also reported as mean ± standard error.

### Histopathology

Muscle tissues inoculated with heat-killed or live bacteria were fixed overnight in 4% paraformaldehyde before being sequentially dehydrated and embedded in paraffin. Paraffin embedded tissues were sectioned on a microtome at 4 µm and stained with hematoxylin and eosin or Gram stain.

## Electronic supplementary material

Supplementary Information

## References

[1] Baker JC, Demertzis JL, Rhodes NG, Wessell DE, Rubin DA (2012). Diabetic musculoskeletal complications and their imaging mimics. Radiographics: a review publication of the Radiological Society of North America, Inc.

[2] Stacy, G. S. & Kapur, A. Mimics of bone and soft tissue neoplasms. *Radiologic clinics of North America* **49**, 1261–1286, vii, doi:10.1016/j.rcl.2011.07.009 (2011). 10.1016/j.rcl.2011.07.009 22024298

[3] Palestro CJ (2015). Radionuclide imaging of osteomyelitis. Seminars in nuclear medicine.

[4] Gafter-Gvili, A. *et al*. [18F]FDG-PET/CT for the diagnosis of patients with fever of unknown origin. *QJM: monthly journal of the Association of Physicians*, doi:10.1093/qjmed/hcu193 (2014). 10.1093/qjmed/hcu193 25208896

[5] van Oosten M (2015). Targeted imaging of bacterial infections: advances, hurdles and hopes. FEMS microbiology reviews.

[6] Becker, W. *et al*. Rapid imaging of infections with a monoclonal antibody fragment (LeukoScan). *Clinical orthopaedics and related research*, 263–272 (1996). 10.1097/00003086-199608000-00033 8769461

[7] Ebenhan T, Gheysens O, Kruger HG, Zeevaart JR, Sathekge MM (2014). Antimicrobial peptides: their role as infection-selective tracers for molecular imaging. BioMed research international.

[8] Fuster D (2011). Usefulness of 99mTc-ciprofloxacin scintigraphy in the diagnosis of prosthetic joint infections. Nuclear medicine communications.

[9] Bettegowda C (2005). Imaging bacterial infections with radiolabeled 1-(2′-deoxy-2′-fluoro-beta-D-arabinofuranosyl)-5-iodouracil. Proceedings of the National Academy of Sciences of the United States of America.

[10] Hernandez FJ (2014). Noninvasive imaging of Staphylococcus aureus infections with a nuclease-activated probe. Nature medicine.

[11] Weinstein EA (2014). Imaging Enterobacteriaceae infection *in vivo* with 18F-fluorodeoxysorbitol positron emission tomography. Science translational medicine.

[12] Bardhan NM, Ghosh D, Belcher AM (2014). Carbon nanotubes as *in vivo* bacterial probes. Nature communications.

[13] Bugg TD, Walsh CT (1992). Intracellular steps of bacterial cell wall peptidoglycan biosynthesis: enzymology, antibiotics, and antibiotic resistance. Natural product reports.

[14] Lam H (2009). D-amino acids govern stationary phase cell wall remodeling in bacteria. Science.

[15] Hammes WP, Neuhaus FC (1974). On the mechanism of action of vancomycin: inhibition of peptidoglycan synthesis in Gaffkya homari. Antimicrobial agents and chemotherapy.

[16] Caparros M, Pisabarro AG, de Pedro MA (1992). Effect of D-amino acids on structure and synthesis of peptidoglycan in Escherichia coli. Journal of bacteriology.

[17] Siegrist MS (2013). (D)-Amino acid chemical reporters reveal peptidoglycan dynamics of an intracellular pathogen. ACS chemical biology.

[18] Kuru E, Tekkam S, Hall E, Brun YV, Van Nieuwenhze MS (2015). Synthesis of fluorescent D-amino acids and their use for probing peptidoglycan synthesis and bacterial growth *in situ*. Nature protocols.

[19] Mizuno KI, Yamazaki S, Iwata R, Pascali C, Ido T (1993). Improved Preparation of L-[Methyl-C-11]Methionine by Online [C-11] Methylation. Appl Radiat Isotopes.

[20] Kaper JB, Nataro JP, Mobley HL (2004). Pathogenic Escherichia coli. Nature reviews. Microbiology.

[21] Tong SY, Davis JS, Eichenberger E, Holland TL, Fowler VG (2015). Staphylococcus aureus infections: epidemiology, pathophysiology, clinical manifestations, and management. Clin Microbiol Rev.

[22] Yang D (1993). Synthesis of [18F]fluoroalanine and [18F]fluorotamoxifen for imaging breast tumors. Journal of drug targeting.

[23] Wang L (2012). Synthesis and evaluation of 18F labeled alanine derivatives as potential tumor imaging agents. Nuclear medicine and biology.

[24] Fasth KJ, Antoni G, Langstrom B (1988). Asymmetric-Synthesis of L-[3-C-11] Alanine and L-[3-C-11] Phenylalanine by a Phase-Transfer Alkylation Reaction. J Chem Soc Perk T.

[25] Krasikova RN (2011). 4-[F-18]Fluoroglutamic Acid (BAY 85-8050), a New Amino Acid Radiotracer for PET Imaging of Tumors Synthesis and *in Vitro* Characterization. Journal of medicinal chemistry.

[26] Koglin N (2011). Specific PET Imaging of x(C)(-) Transporter Activity Using a F-18-Labeled Glutamate Derivative Reveals a Dominant Pathway in Tumor Metabolism. Clinical Cancer Research.

[27] Langstrom B (1987). Synthesis of L- and D-[methyl-11C]methionine. Journal of nuclear medicine: official publication, Society of Nuclear Medicine.

[28] Pascali C (1999). High efficiency preparation of L-[S-methyl-C-11]methionine by on-column [C-11]methylation on C18 Sep-Pak. J Labelled Compd Rad.

[29] Gowrishankar G (2014). Investigation of 6-[(1)(8)F]-fluoromaltose as a novel PET tracer for imaging bacterial infection. PloS one.

[30] Namavari M, Gowrishankar G, Hoehne A, Jouannot E, Gambhir SS (2015). Synthesis of [(1)(8)F]-labelled maltose derivatives as PET tracers for imaging bacterial infection. Molecular imaging and biology: MIB: the official publication of the Academy of Molecular Imaging.

[31] Ning X (2014). PET imaging of bacterial infections with fluorine-18-labeled maltohexaose. Angewandte Chemie.

[32] Pidgeon SE (2015). Metabolic Profiling of Bacteria by Unnatural C-terminated D-Amino Acids. Angewandte Chemie.

[33] Cho I, Blaser MJ (2012). The human microbiome: at the interface of health and disease. Nat Rev Genet.

[34] Comar D, Cartron J, Maziere M, Marazano C (1976). Labelling and metabolism of methionine-methyl-11 C. Eur J Nucl Med.

