# Supplementary material for: Imaging Active Infection in vivo Using D-Amino Acid Derived PET Radiotracers
Source: Sci Rep. 2017 Aug 11;7:7903. doi: 10.1038/s41598-017-08415-x (PMC5554133; doi:10.1038/s41598-017-08415-x)
Supplement: Supplementary file 1 — Supplementary Information [file 41598_2017_8415_MOESM1_ESM.doc]

**Imaging Active Infection *in vivo* Using D-Amino Acid Derived PET Radiotracers**

Kiel D. Neumann^1*^, Javier E. Villanueva-Meyer^1*^, Christopher A. Mutch^1^, Robert R. Flavell^1^, Joseph E. Blecha^1^, Tiffany Kwak^1^, Renuka Sriram^1^, Henry F. VanBrocklin^1^, Oren S. Rosenberg^2^, Michael A. Ohliger^1,3^, David M. Wilson^1^

**Supplementary Information:**


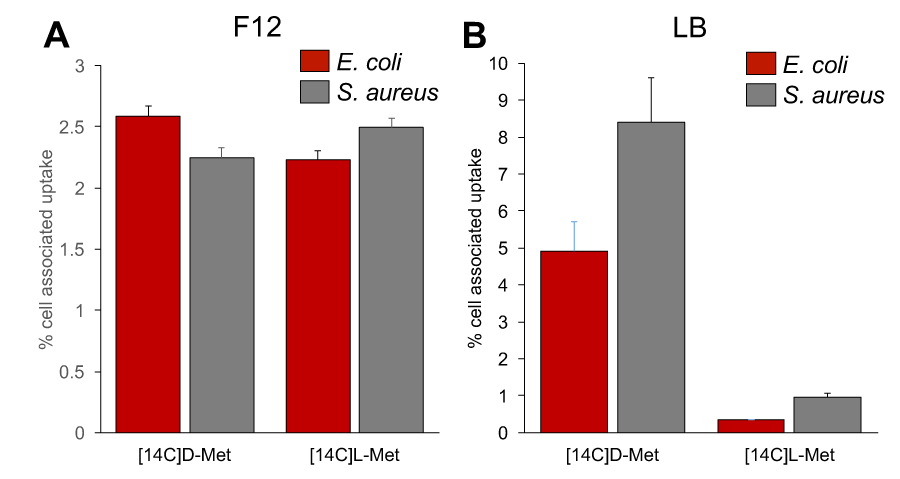


**Supplementary Figure 1.** *In vitro* studies comparing [^14^C] D-Met and [^14^C] L-Met accumulation in *E. coli* and *S. aureus* after 1 hour of incubation (n=4 per study). (a) No statistically significant differences were observed in F12 media. (b) Both *E. coli* and *S. aureus* showed preference for [^14^C] D-Met over [^14^C] L-Met in lysogeny broth (LB).


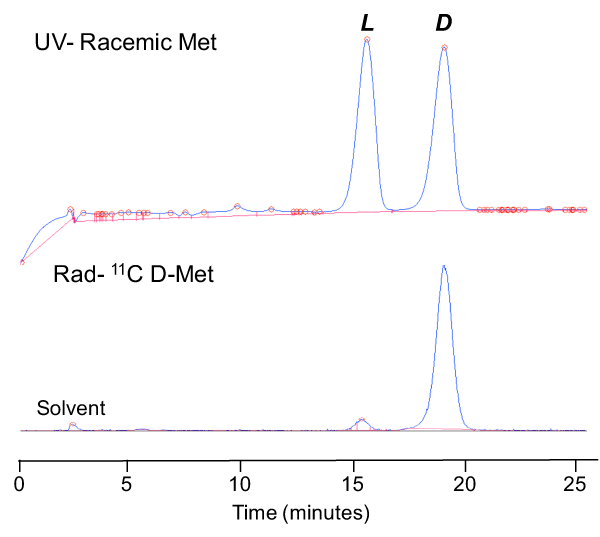


**Supplementary Figure 2.** HPLC analysis of [^11^C] D-Met. All studies used a Phenomenex Chirex-33126 D-penicillamine HPLC column (4.6 x 250 mm) with a mobile phase of 30:70 methanol:1mM copper sulfate at a flow rate of 1 mL/min. (a) HPLC trace of racemic Met standard showing 50/50 L-Met/D-Met. (b) Radio-HPLC of [^11^C] D-Met. Integration showed > 90% D-Met enantiomer.


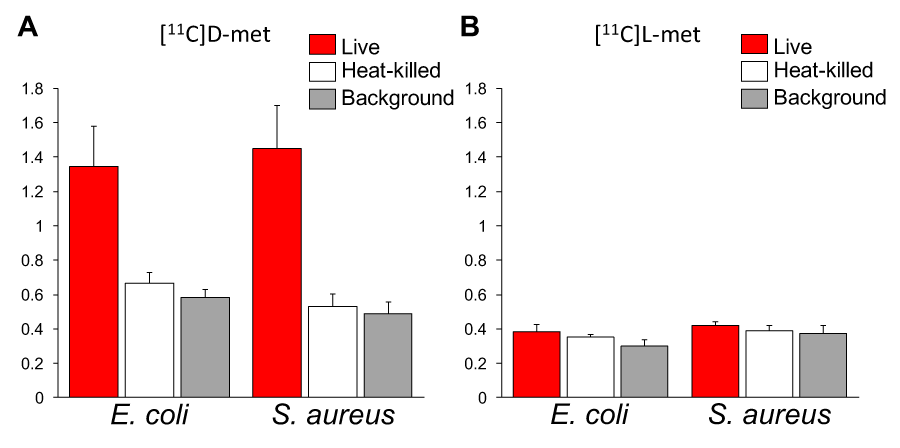


**Supplementary Figure 3.** Raw region of interest (ROI) data obtained for PET images (not background corrected), n=4 in all cases. (a) For [^11^C] D-Met there was > 2-fold increased signal in tissue inoculated with live bacteria, versus the heat-killed side (p < 0.05). The signal from tissue containing heat-killed bacteria was similar to that seen in normal muscle. (b) For [^11^C] L-Met there were no statistically significant differences between ROI’s corresponding to live bacteria, heat-killed bacteria, or normal muscle (p > 0.05 in all cases).


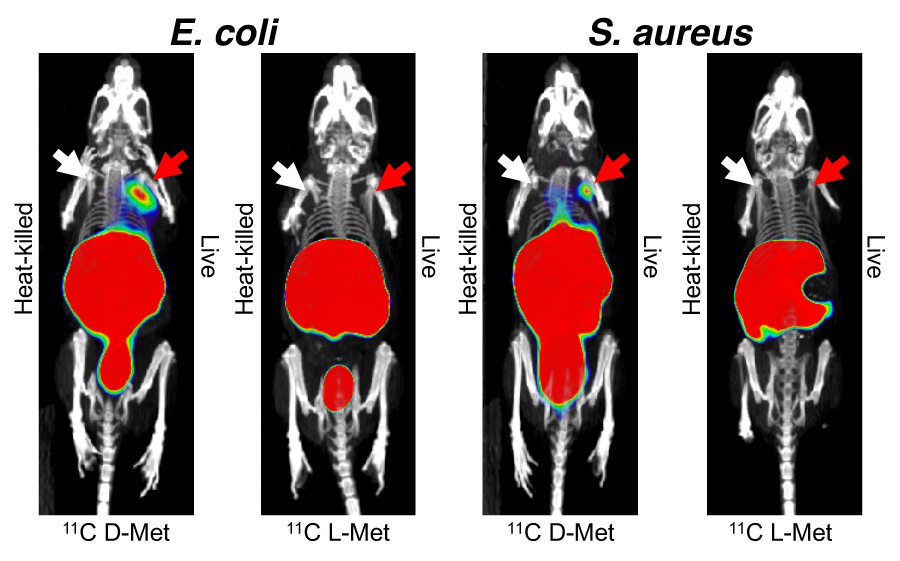


**Supplementary Figure 4.** Maximum intensity projection (MIP) images of [^11^C] D-Met in *E. coli* and *S. aureus* infected mice. Specific accumulation of tracer in living bacteria-inoculated deltoid muscle is seen, as well as significant uptake in liver, kidneys and bladder as confirmed by biodistribution experiments.


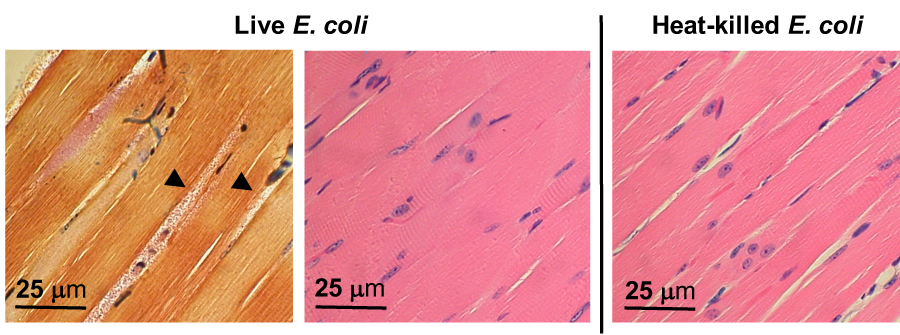


**Supplementary Figure 5.** Representative histology for an *E. coli* infected mouse sampled from deltoid muscle, using Gram-staining (left image) and hematoxylin & eosin (H&E, center and right image). In live inoculations, scattered inflammatory cells and intact bacteria are seen, denoted by arrowheads. In heat-killed inoculations (right), several inflammatory cells are present on H&E staining without discernible bacteria. Images are representative of four animals.


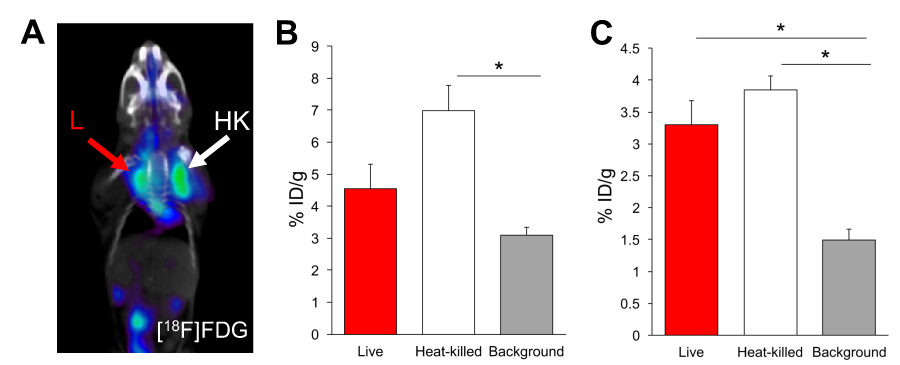


**Supplementary Figure 6.** FDG analysis of a *S. aureus* infected mouse cohort (n=4). Static images were acquired from 45-60 minutes similar to clinical protocols (a) Representative mouse image showing accumulation of tracer in both live bacteria inoculated (L, red arrow) and heat-killed bacteria inoculated (HK, white arrow) muscle over background. In this image the accumulation of FDG appears greater on the heat-killed side, but this difference was not statistically significant over 4 mice. (b) Raw region of interest (ROI) data showed increased signal in muscle inoculated with heat-killed bacteria over normal muscle (p < 0.05). (c) Biodistribution analysis of affected muscle using a gamma-counter showed no significant difference in tracer accumulation between live bacteria inoculated and heat-killed bacteria inoculated muscle (p > 0.05). In contrast, both showed higher uptake than normal muscle (p < 0.05).

| **Trial** | **% RCY (EOB)** | **%D-enantiomer** | **Thiolactone (mg)** | **mM NaOH*** | **Acetone (μL)** | | **10 mM AcOH (μL)** |
| --- | --- | --- | --- | --- | --- | --- | --- |
| 1 | 19 | 80 | 0.2 | 32 | 1000 | 320 | |
| 2 | 8 | 89 | 0.2 | 32 | 500 | 320 | |
| 3 | 28 | 85 | 0.2 | 32 | 500 | 320 | |
| 4 | 17 | 90 | 0.2 | 32 | 500 | 320 | |
| 5 | 20 | 97 | 0.2 | 20 | 500 | 320 | |
| 6 | 15 | 96 | 0.2 | 22 | 500 | 320 | |
| 7 | 39 | 80 | 0.2 | 32 | 500 | 320 | |

**Supplementary Table 1.** Summary of radiochemical synthesis trials for optimization of radiochemical yield (RCY) and % D-enantiomer for [^11^C] D-Met. Entries 5 and 6 show conditions resulting in the highest enantiomeric excess (approx. 92% ee or 96%-D, 4%-L). *This is the NaOH concentration in a 100 μL aliquot used to dissolve the thiolactone precursor.
